# *Yersinia pestis* DNA Sequences in Late Medieval Skeletal Finds, Bavaria

**DOI:** 10.3201/eid1611.100598

**Published:** 2010-11

**Authors:** Ingrid Wiechmann, Michaela Harbeck, Gisela Grupe

**Affiliations:** Author affiliations: Ludwig Maximilian University of Munich, Munich, Germany (I. Wiechmann, G. Grupe);; Bavarian State Collection of Anthropology and Palaeoanatomy, Munich (M. Harbeck, G. Grupe)

**Keywords:** Bacteria, vector-borne infections, zoonoses, Yersinia pestis, Black Death, plague, Middle Ages, Bavaria, archeology, letter

**To the Editor**: *Yersinia pestis*, the causative agent of plague, is held responsible for 3 human pandemics: the Justinian plague (5th–7th century), the Black Death (13th–15th century), and modern plague (1870s to present). In 1894, Alexandre Yersin identified *Y. pestis* during an epidemic of plague in Hong Kong (*1*). However, whether *Y. pestis* was indeed responsible for the medieval epidemic is still controversial. *Y. pestis*­­ specific DNA has been detected in medieval skeletal finds (*2*,*3*), although some investigators have failed to do so (*4*), leading to the suggestion that a viral hemorrhagic fever was the agent of these medieval pandemics (*5*).

Against this background, we investigated a mass burial site that was discovered under the sacristy of the St. Leonhard Catholic church in Manching-Pichl, near Ingolstadt in Bavaria, Germany. In 1984, during the renovation of this church, 75 human skeletons and several scattered skeletal elements were discovered under the sacristy. The skeletons lay densely packed in 4 layers. Apparently, no grave pit had been dug at all, but the bodies were obviously deposited side by side and covered with dirt layer by layer. An approximation of the time of burial was possible only by means of accompanying building structures, which were dated to the Gothic period (1250–1500 ce).

In the course of research preceding this study, our research group found *Y. pestis* DNA in 10 of 33 examined individual skeletal remains from the mass grave beneath the sacristy (*6*) by using the primer pair YP12D/YP11R (*3*). In the current study, the remains of 6 persons from the mass burial site that had positive *Y. pestis* DNA results before were further investigated. DNA from additional tooth samples of these persons was extracted, and more markers mapped on the *Y. pestis* high copy number plasmid pPCP1 were included. In addition to the primer systems YP12D/YP11R and YP11D/YP10R (*3*), the following primer systems were used. The primer pair YP14F (5′-TCCGGGTCAGGTAATATGGA-3′)/YP13R (5′-ACCAGCCTTTCACATTGAGG-3′) amplifies another sequence section (positions 6953–7082, reference: *Y. pestis* strain CO92 plasmid pPCP1 sequence AL109969.1) on the *Y. pestis pla* gene (encoding plasminogen activator). The plasminogen activator belongs to the virulence factors of *Y. pestis*. The primer pair pst-F (5′-GGTAAATCGCTGAACCGAAG-3′)/pst-R (5′-AACAGCACCTCTGACGCTTT-3′) amplifies a 129-bp sequence section (positions 5026–5154, GenBank accession no. AL109969.1) on the *pst* gene (encoding pesticin activity protein). Pesticin is a bacteriocin that is active against only a few closely related microorganisms. The primer pair PCP-F (5′-CATCCACATGCTCAACCCTA-3′)/PCP-R (5′-CTGAACGCATTTCAGTGGTG-3′) can amplify a 128–130-bp sequence section (positions 8428–8555, GenBank accession no. AF053945.1; positions 8428–8557, GenBank accession no. AL109969.1) on plasmid pPCP1.

These 3 new primer sets, designed with the aid of the software component Primer3 (*7*), were applied by using a suicide PCR method (*3*,*8*); i.e., new primer pairs targeting sequences not previously amplified in the laboratory were used. At no time during all examinations was any modern *Y. pestis* DNA included.

The sample preparation, DNA extraction, PCR setup, electrophoretic separation, and sequencing of amplicons are described elsewhere (*6*,*9*); however, we used 0.20 µmol/L of each primer, an annealing temperature of 54°C, and 45 amplification cycles. PCR blanks containing all reagents except for DNA and extraction blanks were included in every PCR set.

Results of the amplification reactions are listed in the Table. All accompanied extraction and PCR controls remained free of amplification products. All amplicons resulting from suicide PCRs were sequenced. Amplicons resulting from the use of primer pairs YP14F/YP13R and pst-F/pst-R matched the reference sequence to 100% (GenBank accession no. AL109969.1). Amplicons resulting from the use of primer pair PCP-F/PCP-R matched this reference sequence to only 97.78%. This deviation is because of a 2-bp insertion (2 Ts, positions 8531 and 8532, GenBank accession no. AL109969.1) at *Y. pestis* strain CO92 plasmid pPCP1. The sequences obtained from 3 persons’ remains showed in the pPCP1 sequence section between nucleotide positions 8528–8532 only 3 Ts instead of 5 Ts described for *Y. pestis* strain CO92 plasmid pPCP1 (GenBank accession no. AL109969.1). The sequences found in this study were deposited in GenBank under accession nos. HQ290521–HQ290523.

**Table:** Amplification results obtained from the skeletal remains of 6 persons excavated at the St. Leonhard Catholic church, Manching-Pichl, Bavaria, Germany*

Identification no.	Sex, age at death, y/sex	Tooth sample	DNA extract†	YP12D/ YP11R‡		YP11D/ YP10R	YP14F/ YP13R	pst-F/ pst-R	PCP-F/ PCP-R
17-I	≈11/M	1st	I	−	+	NT	NT	−	−
		1st	II	−	−	NT	−	NT	NT
		2nd	I	−	−	−	−	NT	NT
22	20–22/F	2nd	I	−	−	−	NT	NT	NT
34-I	Young adult/M	1st	I	+	+	NT	NT	+	+
73-I	20–25/M	1st	I	(+)	(+)	NT	NT	NT	NT
		1st	II	−	−	−	−	NT	NT
		2nd	I	−	−	−	−	NT	NT
S1-I	≈8/M	1st	I	+	+	NT	NT	+	+
S4-XX	≈15/F	1st	I	+	+	NT	NT	+	−
		1st	II	+	+	+	+	+	+
		2nd	I	+	+	+	+	+	+

*Extraction controls and PCR controls had no amplicons. +, amplicon; (+), weak amplicon; –, no amplicon; NT, not tested. †I and II represent separate extracts from the same tooth. ‡Independent PCRs with the primer pair.

To conclude, the successful recovery of several *Y. pestis* plasmid pPCP1 DNA sequences in skeletal finds from the mass burial site excavated in Manching-Pichl suggests that these persons died of plague. Moreover, our findings constitute a molecularly supported confirmation for the presence of *Y. pestis*, the etiologic agent of plague, in late medieval (1250–1500 ce) southern Germany. In future studies, we will attempt to recover chromosomal *Y. pestis* DNA from the mass grave skeletal remains to obtain clues as to the specific *Y. pestis* strain and the microbiology of past plague in Europe.

## References

[1] Yersin A. La peste bubonique à Hong Kong. Ann Inst Pasteur (Paris). 1894;8:662–7.

[2] Drancourt M, Aboudharam G, Signoli M, Dutour O, Raoult D. Detection of 400-year-old *Yersinia pestis* DNA in human dental pulp: an approach to the diagnosis of ancient septicemia. Proc Natl Acad Sci U S A. 1998;95:12637–40. 10.1073/pnas.95.21.12637 9770538 PMC22883

[3] Raoult D, Aboudharam G, Crubezy E, Larrouy G, Ludes B, Drancourt M. Molecular identification by “suicide PCR” of *Yersinia pestis* as the agent of medieval Black Death. Proc Natl Acad Sci U S A. 2000;97:12800–3. 10.1073/pnas.220225197 11058154 PMC18844

[4] Gilbert MTP, Cuccui J, White W, Lynnerup N, Titball RW, Cooper A, Absence of *Yersinia pestis*–specific DNA in human teeth from five European excavations of putative plague victims. Microbiology. 2004;150:341–54. 10.1099/mic.0.26594-0 14766912

[5] Duncan CJ, Scott S. What caused the Black Death? Postgrad Med J. 2005;81:315–20. 10.1136/pgmj.2004.024075 15879045 PMC1743272

[6] Garrelt C, Wiechmann I. Detection of *Yersinia pestis* DNA in early and late medieval Bavarian burials. In: Grupe G, Peters J, editors. Decyphering ancient bones; the research potential of bioarchaeological collections. Documenta Archaeobiologiae; Bd.1. Leidorf (Germany): Rahden/Westf.: 2003. p. 247–54.

[7] Rozen S, Skaletsky H. Primer3 on the WWW for general users and for biologist programmers. In: Krawetz S, Misener S, editors. Bioinformatics methods and protocols: methods in molecular biology. Totowa (NJ): Humana Press; 2000. p. 365–86. 10.1385/1-59259-192-2:365 10547847

[8] Drancourt M, Signoli M, Dang LV, Bizot B, Roux V, Tzortzis S, *Yersinia pestis* Orientalis in remains of ancient plague patients. Emerg Infect Dis. 2007;13:332–3. 10.3201/eid1302.060197 17479906 PMC2725862

[9] Wiechmann I, Grupe G. Detection of *Yersinia pestis* DNA in two early medieval skeletal finds from Aschheim (Upper Bavaria, 6th century A.D.). Am J Phys Anthropol. 2005;126:48–55. 10.1002/ajpa.10276 15386257

